# Plantar flexion force induced by amplitude-modulated tendon vibration and associated soleus V/F-waves as an evidence of a centrally-mediated mechanism contributing to extra torque generation in humans

**DOI:** 10.1186/1743-0003-10-32

**Published:** 2013-03-25

**Authors:** Fernando Henrique Magalhães, Diana Rezende de Toledo, André Fabio Kohn

**Affiliations:** 1 Neuroscience Program and Biomedical Engineering Laboratory, Universidade de São Paulo, EPUSP, Avenida Professor Luciano Gualberto, Travessa 3, n.158, São Paulo, SP, Brazil; 2 School of Arts, Sciences and Humanities - Universidade de São Paulo, EACH-USP, São Paulo, Brazil

**Keywords:** Persistent inward currents, Percussion, Muscle, Vibratory stimulation, V-wave

## Abstract

**Background:**

High-frequency trains of electrical stimulation applied over the human muscles can generate forces higher than would be expected by direct activation of motor axons, as evidenced by an unexpected relation between the stimuli and the evoked contractions, originating what has been called “extra forces”. This phenomenon has been thought to reflect nonlinear input/output neural properties such as plateau potential activation in motoneurons. However, more recent evidence has indicated that extra forces generated during electrical stimulation are mediated primarily, if not exclusively, by an intrinsic muscle property, and not from a central mechanism as previously thought. Given the inherent differences between electrical and vibratory stimuli, this study aimed to investigate: (a) whether the generation of vibration-induced muscle forces results in an unexpected relation between the stimuli and the evoked contractions (i.e. extra forces generation) and (b) whether these extra forces are accompanied by signs of a centrally-mediated mechanism or whether intrinsic muscle properties are the predominant mechanisms.

**Methods:**

Six subjects had their Achilles tendon stimulated by 100 Hz vibratory stimuli that linearly increased in amplitude (with a peak-to-peak displacement varying from 0 to 5 mm) for 10 seconds and then linearly decreased to zero for the next 10 seconds. As a measure of motoneuron excitability taken at different times during the vibratory stimulation, short-latency compound muscle action potentials (V/F-waves) were recorded in the soleus muscle in response to supramaximal nerve stimulation.

**Results:**

Plantar flexion torque and soleus V/F-wave amplitudes were increased in the second half of the stimulation in comparison with the first half.

**Conclusion:**

The present findings provide evidence that vibratory stimuli may trigger a centrally-mediated mechanism that contributes to the generation of extra torques. The vibration-induced increased motoneuron excitability (leading to increased torque generation) presumably activates spinal motoneurons following the size principle, which is a desirable feature for stimulation paradigms involved in rehabilitation programs and exercise training.

## Background

High frequency trains of electrical stimulation applied over the muscles can generate forces that are larger than would be expected by direct activation of motor axons, as evidenced by an unexpected relation between the stimuli and the evoked contractions [1-3]. These extra torques/forces were not present when a nerve block was applied proximal to the stimulation site [1,2,4], but remained present both in complete spinal cord-injured [1,5] and healthy sleeping subjects [1], suggesting that an involuntary, centrally-mediated mechanism (i.e. generated by intrinsic properties of spinal neurons) was behind the extra torque generation. More specifically, the extra torque was proposed to be due to an increase in firing rate and recruitment of new motoneurons through either the activation of persistent inward currents (PICs) and/or the development of post-tetanic potentiation (PTP)^a^[1,2].

However, in an elegant series of experiments performed in humans and in decerebrate cats, Frigon et al. [6] have provided strong evidence that the extra torque generated during electrical stimulation is mediated primarily, if not exclusively, by an intrinsic muscle property (i.e. a peripheral mechanism), and not from a central mechanism as previously thought. PIC amplitudes are known to be modulated by limb position, with larger PIC amplitudes at longer muscle lengths [7]. However, Frigon et al. [6] observed that the extra torques generated by electrical stimulation of the ankle muscles increased at shorter muscle lengths, a typical behavior attributed to peripheral muscle properties [8,9]. Moreover, extra forces and their length-dependent modulation were unaffected by sciatic nerve transection or methoxamine injection in cats and nerve block in humans [6]. Therefore, whether the electrically-evoked extra forces are generated by nonlinear input/output properties intrinsic to spinal neurons or to the muscle itself is still a controversial issue, with the most recent findings pointing to a mechanism that is intrinsic to the muscle.

It is well established that motoneuron firing is modulated by intrinsic motoneuron properties and by synaptic inputs. For example, the activation of PICs requires the opening of voltage-gated L-type Ca ++ channels in the motoneuron dendrites, which occurs due to summation of excitatory postsynaptic potentials. These PICs may produce continuous depolarization (plateau potential) [10,11] and consequently self-sustained motoreuron discharge. A transient depolarization of sufficient amplitude and duration (“on” stimulus) can initiate a plateau potential [12]. For example, high-frequency (300 Hz) electrical stimulation of afferent fibers can activate PICs in the associated motoneurons of decerebrate cats [13]. Similarly, synaptic inputs from Ia afferents has been shown to generate PICs that are distributed extensively throughout the motoneuron pool of decerebrate cats [13-17]. However, the extra torques evoked by high-frequency stimulation may not reflect univocally what occurs at the motoneuron, but may be due to peripheral muscle properties, at least with regard to muscle torques/forces evoked by electrical stimulation [6]. Therefore, the questions that may be posed are whether extra torques may be evoked by high-frequency *vibration* and whether these extra torques may be due to peripheral muscle properties or central neural mechanisms.

High frequency (e.g., 80 Hz or higher) vibratory stimulation of a tendon mainly stimulates muscle spindle primary endings [18,19] and may evoke involuntary repeated muscle contractions via tonic vibration reflexes (TVRs) [20]. The complex action of vibration on the motoneuron includes both monosynaptic and oligosynaptic excitation [21], and the activation of plateau potentials has also been suggested to play an important role in the generation of TVR in the unanesthetized decerebrate cat [22]. However, direct experimental evidence that vibratory stimuli facilitate the genesis of an intrinsic motoneuron property (i.e. PICs activation) in humans has been obtained only at a low level of muscle activation that involved paired motor unit recordings [23-25]. For example, Gorassini et al. [23] showed that brief vibration can lead to self-sustained firing in human motoneurons, as vibratory stimulation of the tibialis anterior tendon recruited an additional motor unit (i.e. “test” unit) even though the firing rate of the “control” unit (firing due to the maintenance of a low-level background voluntary contraction) remained the same or decreased.

Whether a centrally-mediated mechanism (e.g. self-sustained firing of motoneurons) may or may not play a role in the generation of extra forces evoked by vibration is not yet clear from the data found in the literature. In other words, it is unknown whether the strong muscle contractions observed in response to vibratory stimulation (TVRs) reflects a contribution of a nonlinear input/output property intrinsic to spinal neurons. Furthermore, paradigms avoiding voluntary contractions might be preferable, so as to exclude the influence of possible changes in cortical excitability. Therefore, the aim of the present study was to investigate: (a) whether the generation of vibration-induced large muscle forces would result in an unexpected relation between the stimuli and the evoked contractions (i.e. extra torque/force generation) and (b) whether these extra torques would be accompanied by signs of a centrally mediated mechanism (e.g. PICs activation) or whether intrinsic muscle properties would be the most likely mechanisms.

It should be emphasized that there are important differences between the effects of electrical and vibratory stimuli. An obvious difference is the lack of antidromic activation of motoneuron (and sensory) axons during vibration. This means that there is no collision (and annihilation) of reflexively generated action potentials and the antidromic action potentials. In addition, the temporal dispersion of Ia afferent volleys in the tibial nerve induced by Achilles tendon percussion is much greater than that of electrically induced volleys, which may lead to differences in central transmission [26]. Furthermore, group II, Ib and cutaneous afferent discharges induced by electrical stimulation of the tibial nerve are different from those induced by Achilles tendon percussion [27,28]. Hence, whether substantial levels of muscle forces evoked by vibration are mediated by properties intrinsic to motoneurons and/or to the muscle cannot be predicted from the previous experiments with electrical stimulation.

In the present paper, plantar flexion torque (i.e. triceps surae muscle force) is measured in response to a 100 Hz vibration whose amplitude is slowly increased and then decreased back to zero. This paradigm was motivated by previous experiments in animals where the development of plateau potentials in motoneurons resulted in a marked hysteresis in the input–output properties of the cell (current–voltage, current-frequency) during triangular-shaped patterns of electrical stimulation [17]. Similarly, electrical stimulation patterns with the frequency modulated by a triangular waveform (linearly increasing to a peak frequency and then linearly decreasing) produced greater muscle force on the decreasing side of the ramp. This result could be due to plateau potential generation [1,2], but it may also be due to peripheral properties intrinsic to the muscle [29].

As a measure of motoneuron excitability estimated at different times during the vibratory stimulation, short-latency compound muscle action potentials (CMAPs) were recorded in the soleus muscle in response to supramaximal stimulation delivered to the posterior tibial nerve. In the present study, such responses are interpreted as V-waves, F-waves, or even a combination of both. Therefore, the term V/F-waves will be used in the text. A more detailed explanation about the generation of V/F-waves is given in the first two paragraphs of the Discussion.

## Methods

Six male subjects (27.5 ± 6.3 years; mean ± SD) volunteered to participate in this study. All subjects were healthy and physically active, with no known musculoskeletal injuries or neurological disorders. All were right-footed. All subjects gave informed consent and all procedures were approved by the Human Ethics Committee of the Institute of Psychology at the University of São Paulo. The experiments were conducted in accordance with the Declaration of Helsinki.

Subjects were seated on a customized chair designed for measuring ankle torque during isolated isometric plantar flexion contraction. The hip, knee and ankle of the right leg were maintained at 90°. A strain gauge force transducer (Transtec N320, Brazil) was attached to the pedal to which the right foot was fastened.

At the beginning of the session, subjects were asked to perform two MVCs of plantar flexion. The maximum force value achieved was taken as the MVC force value. All measurements in this paper are expressed as a percentage of the MVC (and hence the terms torque and force are used interchangeably).

The Achilles tendon of the right leg was stimulated mechanically by means of a LW-126-13 vibration system (Labworks, USA), consisting of a power amplifier and a shaker (cylindrical body, with diameter 10.5 cm and length 13.5 cm). The shaker was positioned so that the tip of the shaker (round-shaped plastic tip, 1 cm diameter) was pressed against the Achilles tendon in order to keep a steady pressure and a fixed position on the tendon. A LabView system (National Instruments, USA) was utilized to generate amplitude-modulated signals with 20-s duration, which were delivered to the input of the shaker’s power amplifier in order to obtain the desired mechanical stimulation. More specifically, 100-Hz sinusoidal signals were modulated in amplitude by a triangular wave of 0.05 Hz, so that the displacement amplitude of the 100-Hz vibrations linearly increased from zero (no stimulation) to a peak (around 5 mm peak-to-peak of the sinusoidal displacement) during 10 s of stimulation and then linearly decreased to zero during other 10 s. An ADXL78 accelerometer (Analog Devices, USA) was attached to the movable part of the shaker in order to monitor the parameters of the mechanical stimuli. The peak-to-peak acceleration of the 100-Hz sinusoidal vibration recorded at time = 10 s of the stimulation signal (i.e. at the peak amplitude of the triangular wave) was 200·g in the average (200 times the acceleration of gravity), which corresponds to a peak-to-peak displacement of the tip of the shaker around 5 mm.

Torque signals were low-pass filtered at 15 Hz using a Butterworth filter. The torque generated in response to each of the 20-s stimulation trial was plotted as a function of time. The area under the torque was then quantified for the first and for the second half of the vibratory stimulation: 0 to 10 s (area = T) and 10 to 20 s (area = T’). Note that T and T’ correspond to the same displacement amplitude of the vibratory stimulation.

Surface EMG signals were recorded using round-shaped surface electrodes (0.8 cm diameter, proximal-distal orientation, with an inter-electrode distance of 2 cm) positioned over the right soleus muscle, the most proximal contact being 4 cm beneath the inferior margin of the two heads of the gastrocnemius muscles. A ground electrode was placed over the tibia. The EMG signals were amplified and filtered (10 Hz to 1 kHz) by a MEB-4200 system (Nihon-Kohden, Japan). For qualitative evaluation, the EMG envelope was obtained by low-pass filtering (5-Hz cut-off frequency) the rectified EMG signals.

V/F-waves were evoked by supramaximal electrical stimulation (test stimuli) of the posterior tibial nerve (duration, 0.2 ms) delivered through surface electrodes with the cathode (area = 2 cm^2^) in the popliteal fossa and the anode (area = 8 cm^2^) against the patella. The maximal peak-to-peak amplitude of the soleus CMAP (maximal M wave, Mmax) was obtained. The stimulus intensity used to elicit V/F-waves was ~150% of that required to elicit the Mmax.

**Figure 1:**
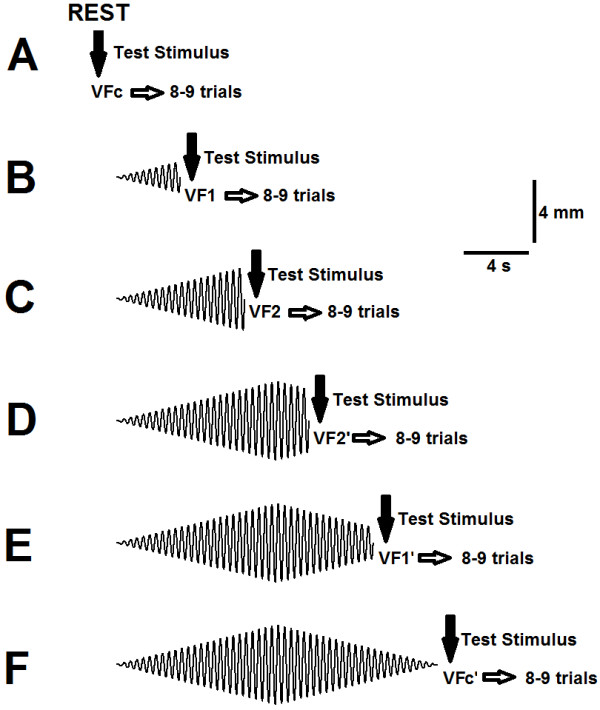
**Schematic representation of the experimental paradigm used to obtain V/F waves. A)** Eight to nine supramaximal stimuli (with at least 1 minute between consecutive stimuli) were delivered to the posterior tibial nerve in order to obtain V/F responses that represent the “control” condition (Fc). **B-E**) A single supramaximal stimulation was delivered at latencies 4 (B, VF1), 8 (C, VF2), 12 (D, VF2^′^) and 16 (E, VF1^′^) seconds after the beginning of the vibratory stimulation. An independent stimulation trial was performed for each V/F-wave, so that 8–9 stimulation trials were performed for each latency in order to obtain the desired number of V/F responses. For all cases, at least one resting minute was allowed between consecutive trials. **F**) Eight to nine supramaximal stimuli (with at least 1 minute between consecutive stimuli) were delivered to the posterior tibial nerve immediately after the entire vibratory stimulation duration (20 s), yielding V/F responses (in the text referred to as VFc’).

A single supraximal stimulus was delivered to the posterior tibial nerve at the latencies 4, 8, 12 and 16 seconds after the beginning of the vibratory stimulation. The test stimulus terminated the stimulation trial, that is, the vibratory stimulation was discontinued after the delivery of a supramaximal stimulus. This avoided the torque generated in response to the test stimulus to interfere with the rest of the stimulation trial and also avoided the influence from sensory receptors activated by the test stimulus. Therefore, an independent stimulation trial was performed for each V/F-wave obtained. A sample of 8 to 9 V/F-waves was obtained for each latency described above, thereby requiring 8–9 stimulation trials to be performed for each latency. The respective responses are named here VF1 (for 4 s latency), VF2 (for 8 s latency), VF2^′^ (for 12 s latency) and VF1^′^ (for 16 s latency). In addition, V/F-waves were also obtained at rest (before vibration initiation) and immediately after the entire vibratory stimulation duration (20 s), named VFc and VFc’, respectively. For all cases, at least one minute of rest was allowed between consecutive trials. Figure 1 illustrates the experimental paradigm used to evoke V/F-waves.

**Figure 2:**
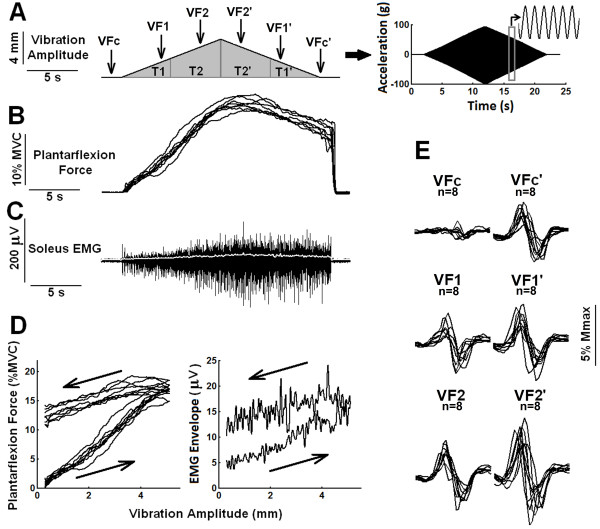
**Plantar flexion force and associated EMG signals and V/F-waves generated by vibratory stimuli modulated in amplitude by a triangular envelope. A**) Schematic representation of the triangular envelope of the vibratory stimulus, showing the time course of a 100-Hz sinusoidal vibration that linearly increased in amplitude from zero to a peak during 10 s of stimulation and then linearly decreased to zero during the next 10 s. The arrows below VFc, VF1, VF2, VF2^′^, VF1^′^ and VFc’ represent the times at which supramaximal electrical stimuli were delivered. T, and T’ represent the torque areas used for data analysis. A representative acceleration signal is shown at the right, with part of the signal (in expanded time scale) in the inset. **B**) Plantar flexion torque (eight superposed recordings) and **C**) EMG from the soleus muscle (typical recording) in response to the stimulation paradigm shown in (**A**). The thin white line in (**C**) is the envelope of the rectified EMG. **D**) The same data shown in (**B**) and (**C**) in graphs of force and EMG envelope as a function of vibration-amplitude (note the marked hysteresis in the force generation and EMG). The increasing and decreasing phase of the vibration amplitude are represented by the arrows pointing to the right and to the left, respectively. **E**) V/F-waves (eight superimposed recordings) obtained at different times during the vibratory stimulation. Note the larger V/F-wave amplitudes during the second half of the stimulation (VF1^′^,VF2^′^) and immediately after the stimulation (VFc’). Data in this figure are from a representative subject that showed clear increases in force, EMG and V/F-wave amplitudes (see also Figure 3) during the second half of the triangular-shaped vibratory stimulation.

The rationale to choose such a paradigm was that V/F-waves would be compared under equivalent displacement amplitudes of the vibratory stimulus, because vibration amplitudes are equivalent at 4 and 16 seconds as well as at 8 and 12 seconds of vibratory stimulation (i.e. around 2 mm for VF1 and VF 1^′^ and around 4 mm for VF 2 and VF 2^′^, while VF c and VF c’ were obtained without stimulation). Areas under the torque curves were quantified as T and T’ (as explained above) using a similar rationale. Figure 2A illustrates the different times at which supramaximal electrical stimuli were delivered and a simple representation of the torque areas is shown.

The subjects were asked to relax completely, not making any voluntary effort during the stimulation trials. Each subject completed 8 to 9 trials of each stimulation paradigm described above. Torque, EMG and acceleration signals were acquired (sampled at 5 kHz).

The peak-to-peak amplitude of each V/F-wave was normalized to the amplitude of its respective Mmax. This is important because variations on Mmax amplitudes with varying levels of voluntary contraction of the soleus muscle have been reported, even though the ankle position (joint angle) remained unchanged [30], which supports previous recommendation [31] that in reflex studies it is necessary to normalize reflex response amplitudes to the corresponding Mmax obtained at the same joint angle and under the same experimental conditions. In order to allow appropriate comparisons, the peak-to-peak amplitudes of the V/F-waves (each expressed as a percentage of its respective Mmax) were normalized by the mean amplitude obtained in the control condition (i.e. at rest, Fc). A repeated-measures ANOVA with planned comparisons [32] was used to compare the V/F-wave peak-to-peak amplitudes between F1 and F1^′^, F2 and F2^′^ and Fc and Fc’. A two-tailed paired *t* test was used to compare the torque areas (expressed in percentage of MVC multiplied by seconds) between T and T’.

Since T and T’ correspond to torque areas taken during the first and the second half of the vibratory stimulation, respectively, we have also performed a paired *t* test in order to compare the amplitude of the V/F-waves taken during the first half of the vibratory stimulation with those taken during the second half. For this analysis, VF1 and VF2 measurements were included in a single sample (VF), the same being done for F1^′^ and F2^′^ (resulting in VF’). Note that by agglutinating V/F-wave measurements, VF and VF’ were composed by samples of 16 to 18 F-waves for each subject, which is approximately two times higher than the sample sizes of VF 1, VF 2, VF 1^′^ and VF 2^′^ (see description above).

All the analyses were performed using the statistical package SPSS 15.0 for Windows (SPSS, Inc., Chicago, IL), with significance level set at P < 0.05.

## Results

**Figure 3:**
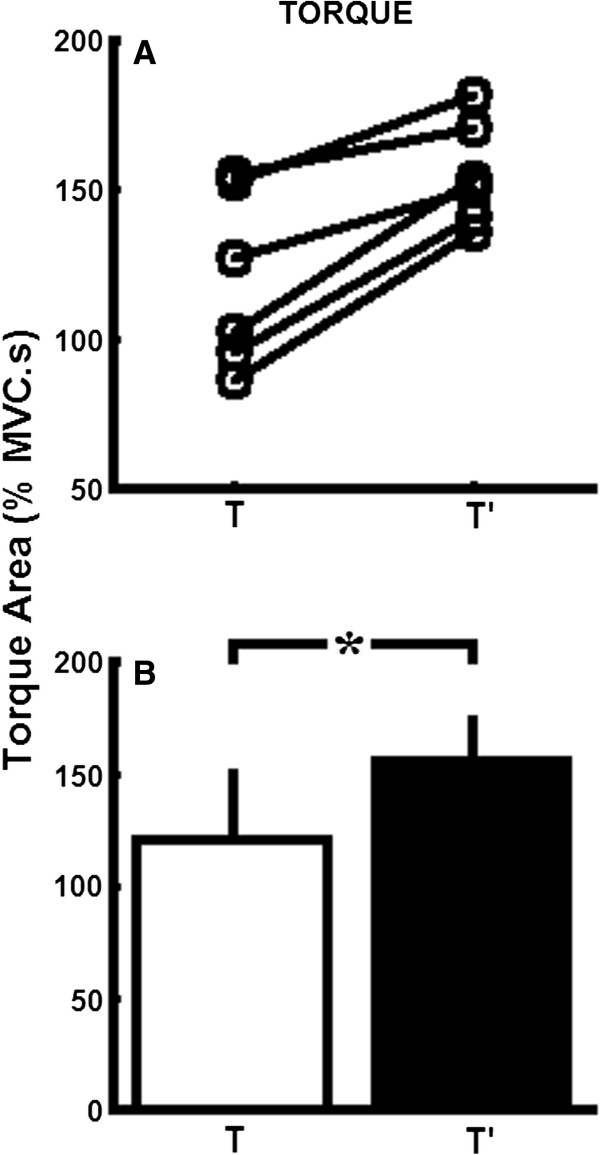
**Torque areas computed for the first (T) and second (T’) half of the vibratory stimulation. A**) Average torque areas calculated for individual subjects, indicating the changes in torque area from T to T’. **B**) Average group data calculated for torque areas. White and black bars represent data obtained during the increasing (T) and decreasing (T’) phase of the vibration amplitude, respectively. Asterisks indicate significant differences (P < 0.05) between conditions.

**Figure 4:**
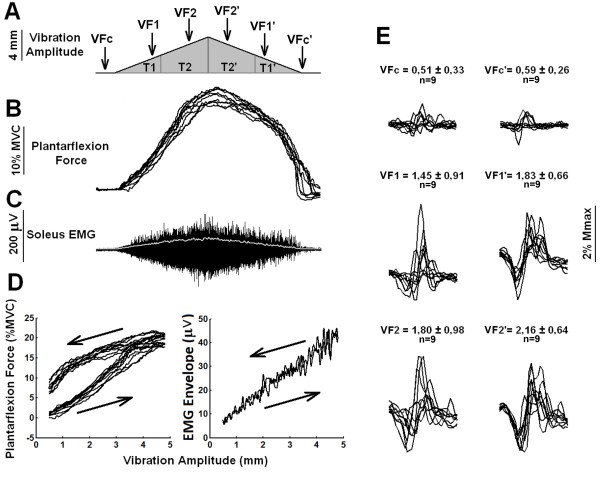
**Plantar flexion force and associated EMG signals and V/F**-**waves generated by vibratory stimuli modulated in amplitude by a triangular envelope.** The same description as in Figure 2 applies to panels A, B, C and D. The numbers above each sample in (**E**) are mean ± SDs of the peak-to-peak V/F-wave amplitudes (n=9), expressed in % of the Mmax. Data in this figure are from a representative subject that showed little evidence of increased motoneuron excitability during the second half of the triangular-shaped vibratory stimulation (see EMG and V/F-wave amplitudes in **D** and **E**, respectively).

Figure 2B-D shows force and EMG signals generated by amplitude-modulated vibratory stimuli for a representative subject. Note that during the decreasing phase of the vibration amplitude (second half of stimulation), the force decayed slower than expected on the basis of the force build up that occurred during the rising vibration amplitude (Figure 2B). The same behavior could be observed with regard to the time course of the EMG build up. This means that there is a marked hysteresis in the force generation (Figure 2D), which was observed for all the 6 subjects analyzed (see Figure 3A). On the other hand, the clear hysteresis shown in the graph of the EMG envelope as a function of vibration amplitude (Figure 2D, right panel) did not happen for all subjects (see Figure 4 for an example in which no clear hysteresis occurred in the EMG envelope *versus* vibration amplitude plot). V/F-waves evoked during the second half of the stimulation showed peak-to-peak amplitudes larger than those obtained during the first half of the stimulation. Figure 2E shows VFc, VFc’, VF, VF1^′^, VF2 and VF2^′^ obtained from a representative subject in which clear increases in V/F-waves amplitudes could be observed (see Figure 4E for a subject that showed a smaller evidence of this behavior).

**Figure 5:**
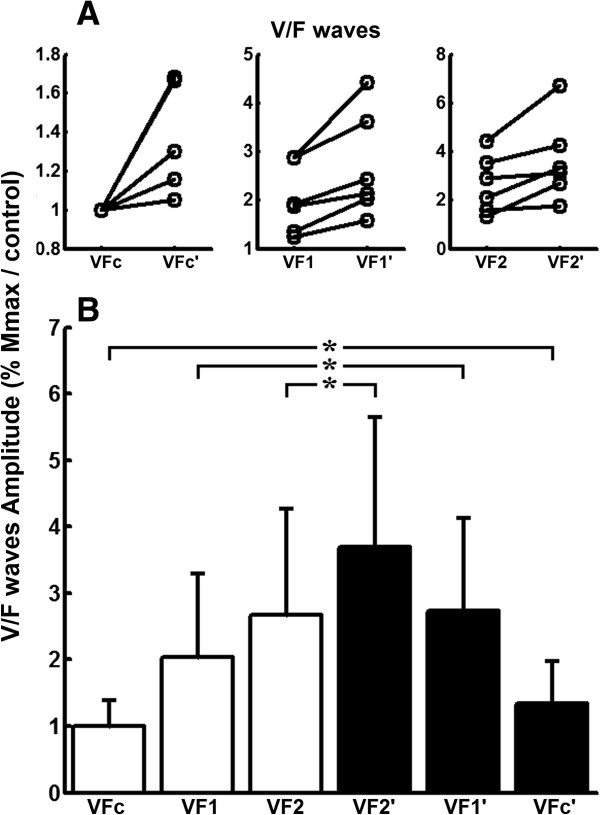
**V/F-wave amplitudes computed at different times during the amplitude-modulated vibratory stimulation. A**) Average V/F-wave amplitudes calculated for individual subjects, indicating the changes from VFc to VFc’, from VF1 to VF1^′^ and from VF2 to VF2^′^. **B**) Average group data calculated for V/F-wave amplitudes.White and black bars represent data obtained during the increasing (VFc, VF1 and VF2) and decreasing (VF1^′^, VF2^′^and VFc’) phase of the vibration amplitude, respectively. Asterisks indicate significant differences (P < 0.05) between conditions.

Comparisons performed on group data (n = 6) showed significant differences between the area under the torque taken during the first (T) and the second (T’) half of the vibratory stimulation (t(5) = 5.824, P = 0.002), as shown in Figure 3. In addition, group data showed significant differences between peak-to-peak V/F-wave amplitudes obtained at different times during the triangular-shaped vibratory stimulation (F(5,30) = 5.571, P = 0.001). Planned comparisons showed that VFc’ was larger than VFc (F(1,5) = 9.002, P = 0.030), VF1^′^ was larger than VF1 (F(1,5) = 12.362, P = 0.017) and VF2^′^ was larger than VF2 (F(1,5) = 9.084, P = 0.029) (Figure 5). Comparisons performed on group data (n = 6) showed significant differences between V/F-wave amplitudes taken during the first (VF) and the second (VF’) half of the vibratory stimulation (t(5) = 7.081, P = 0.001).

## Discussion

Both F and V-waves are routinely obtained in response to strong electrical stimulation (supramaximal stimulation) applied to a peripheral nerve. Such stimulation generates action potentials that travel orthodromically through the efferent fibers and reach the muscle, thereby eliciting a strong, direct motor response (M_MAX_). No H-reflex is observed due to the collision between antidromic and orthodromic spikes. However, the action potentials traveling antidromically reach the cell bodies of the MNs making a small fraction of them to fire orthodromic action potentials that travel back towards the muscle. This generates a relatively small amplitude CMAP called F-wave, which is typically obtained in resting subjects (or at least under the absence of any voluntary effort). On the other hand, if the subject maintains a steady voluntary contraction and the same supramaximal stimulus is delivered to the peripheral nerve, a reflex response appears, called the V-wave (associated with a voluntary drive). The rationale behind the genesis of this response is that the descending drive activates a subset of MNs making their axons conduct action potentials orthodromically. These action potentials collide with the antidromic volley generated by the supramaximal stimulation. Consequently, this subset of MNs (recruited by the descending command) will be susceptible to be activated by the reflex afferent volley generated by the supramaximal electrical stimulus. Computer simulations of a large-scale neuromuscular model [33] provide an illustration of the mechanisms behind the generation of the V-wave [34]. Therefore, F-waves are considered to be a general measure of excitability of the spinal motoneuron pool [35,36] and have been used to assess motoneuron excitability in a large diversity of protocols [37-40], while V-waves roughly reflect the number of spinal MNs being activated by the volitional drive [41,42], and hence are frequently used to measure the level of efferent drive.

In the present study, subjects were relaxed completely, as they were told not to make any voluntary effort during the stimulation trials. Therefore, if all MNs remained silent while the supramaximal test stimulus was applied one would obtain F-waves in response. However, if the vibratory stimulation used in the present study activated a plateau potential mechanism this could make the MNs to discharge autonomously. Therefore, V-waves would occur as a consequence of the collision between action potentials generated by self-sustained MN discharges and those traveling antidromically (i.e. activated by the electrical supramaximal stimulation), thereby allowing the reflexively generated efferent volley to reach the muscle. In this context, V-waves would reflect the number of MNs being activated involuntarily, due to bistable MN behavior, rather than by a volitional drive as in most studies involving V-wave measurements. This is a novel method/interpretation that may be very useful for those researching the occurrence of bistability in human MNs. Actually, the CMAPs recorded in the present study may have resulted from a summation of F-waves and V-waves because not all MNs of a given pool can enter a bistable state involving self-sustained firing [43]. Therefore, this paper refers to V/F waves as the short-latency CMAPs recorded in the soleus muscle in response to supramaximal stimulation to the posterior tibial nerve. Particularly, increased V/F waves represent augmented levels of MN excitability, by reflecting self sustained firing of fully bistable MNs (V-waves) and/or plateau potentials generated in partially bistable MNs (F-waves). For a deeper discussion regarding bistable behavior of spinal MNs, see [10,44].

Higher levels of plantar flexion torque were generated during the second half of the triangular-shaped vibratory stimulation than in the first half. These higher torque values were associated with significant increases in soleus V/F -wave amplitudes, which provide strong evidence that motoneuron excitability was higher during the decreasing amplitude part of the vibratory stimulation. A recent paper has addressed this issue by means of computer simulations of a large-scale neuromuscular model [45]. The simulations showed a similar hysteresis in torque development to a triangular input activation as that found here in human subjects (see their Figure six). More importantly, this hysteresis was only found when the MNs were generating plateau potentials due to PIC generation in their dendrites. The delay in peak torque seen in Figure 2 with respect to the peak of the vibratory input may be attributed to the slow dynamics of plateau potential generation, yielding a slow increase of MN firing rate (see Figure two in [46]).

Additionally, the soleus muscle EMG envelope values were usually increased, probably by a combination of recruitment of additional motor units and increase in the firing rate of already recruited motor units. Both presynaptic and postsynaptic mechanisms may have contributed to these findings. More specifically, the increased force and motoneuron excitability generated during the second half of the stimulation may have been a result of the development of motoneuron plateau potentials (a postsynaptic mechanism) and/or synaptic post-tetanic potentiation (PTP, a presynaptic mechanism consisting of increased motoneuron EPSP amplitudes when a peripheral afferent discharges at a high rate). Both mechanisms could have been triggered by high frequency activation of large sensory afferents from the muscle spindles in response to the high-frequency vibratory stimuli delivered during the first 10 s of stimulation [12,47]. Moreover, PTP may accelerate the development of plateau potential activation due to the increased membrane depolarizations. Computer simulations of the neuromuscular model mentioned above [45] also showed a similar hysteresis that was found here (compare Figure 2C of the present paper and Figure eight left panel in [45]). Hence, again the data are compatible with the development of bistability and plateau potentials in spinal cord MNs.

It is interesting to point out that the facilitation found at the level of the motoneuronal pool in the present experiments occurs despite the possible development of several inhibitory mechanisms. Successive activation of the Ia afferents could have led to postactivation synaptic depression due to reduced neurotransmitter release [47], offsetting (partially or completely) the putative PTP mechanism mentioned above. Ib tendon afferents may have responded to the sinusoidal vibration, even if not in a 1:1 relationship with each cycle [19], which could have activated inhibitory spinal interneurons. Recurrent inhibition from Renshaw cells may also have been involved, since the motoneurons were probably recruited in synchrony with the sinusoidal vibration [48].

Hence, in view of all the issues raised in the last paragraph, the results described in the present paper could not have been easily predicted beforehand, due to the interplay of many putative excitatory and inhibitory mechanisms. Apparently, the effect of plateau potentials (and perhaps PTP) was stronger than those due to inhibitory mechanisms that would have depressed the motoneuron pool excitability. The recent study of Frigon et. al. [6] showed that extra forces evoked during electrical stimulation are primarily mediated by an intrinsic muscle property, since there was no evidence of a centrally mediated mechanism contributing to the extra torque generation. As stated in the Introduction section, there are important differences between the effects of electrical and vibratory stimuli, which are likely to account for the differences between the results and conclusions of the present study and that of Frigon et al. [6].

Qualitatively, a considerable inter-subject variability in the development of increased force and EMG levels and increased V/F -waves was observed, whereas variability within a given subject was much smaller. Between subjects, the responses ranged from little evidence of increased EMGs and V/F-waves to those in which clear increments could be observed. For example, the data shown in Figure 2 are from a representative subject that showed clear increases in plantar flexion torque generation and soleus EMG build up as well as clear increases in V/F-wave amplitudes during the second half of the vibratory stimulation. Figure 4 depicts data from a subject that exhibited a smaller evidence of this behavior (particularly with regard to the EMG build up and V/F-wave amplitudes). This variability may be explained by inter-subject variations in the levels of monoamines such as serotonin and norepinephrine within the spinal cord, known to be related with the development of PICs in animal studies [15]. Interestingly, greater muscle force was observed on the descending slope of the triangular frequency ramps, even in the subjects that showed little evidence of increased EMGs and V/F -waves (Figure 4). This provides evidence that despite the clear contribution from a centrally-mediated mechanism, peripheral properties intrinsic to the muscles also play a role (see below).

Although vibratory stimuli may influence the level of cortical excitability [40], the absence of voluntary contractions in our experimental paradigm makes it less likely that increased motoneuron excitability (as suggested by increases in V/F-wave amplitudes) is influenced by changes in cortical excitability, as could happen during voluntary or electrically-evoked contractions [49]. Furthermore, the absence of voluntary contractions also makes it less likely that intrafusal thixotropy plays a role in the increased torque generation [50] found during the second half of the vibratory stimulation. However, contributions from other peripheral mechanisms such as extrafusal thixotropy, intracellular calcium levels of muscle fibers and muscle potentiation from myosin light chain phosphorylation cannot be excluded and, indeed, there is evidence for a contribution of peripheral mechanisms, as already commented above. But, even with these peripheral mechanisms contributing to the generation of increased forces, there is a clear contribution from a central component, as evidenced by the significant increases in V/F-wave amplitudes.

Muscle stretch lowers the activation threshold of PICs [16,17] while PIC amplitudes in the triceps surae motoneurons (recorded in decerebrated cats) are larger with stretched muscles [7], which may be associated with the TVR facilitation that has been reported at longer muscle lengths [6]. Nevertheless, the present research evidences that a centrally-mediated mechanism contributes to the vibration-induced extra torques, even though the soleus muscle (from which EMG signals were recorded) length used in the present experiments (in a neutral position, i.e. with the ankle joint at 90°) is less likely to reflect central motoneuron properties such as PIC activation than with the muscle stretched. Whether stronger evidence of a centrally-mediated mechanism in the generation of vibration-induced extra torques occurs at longer muscle lengths (changing the angle of the ankle) is a question to be investigated in future research. Furthermore, in addition to the amplitude-modulated paradigm presented here, it would be interesting to see the effects of frequency-modulated vibration on muscle force and CMAP amplitudes, which, technically speaking, represents a challenge since the amplitude of the frequency-modulated sinusoidal signals (i.e. the input to the electrical–mechanical transducer) would have to change in a specific way with the varying frequency in order to yield a constant vibration amplitude at the shaker’s output.

Magalhães and Kohn [51] showed that alternated patterns of high-frequency vibratory bursts with lower frequency trains of electrical stimulation generated increased torques that were accompanied by increased soleus F-wave amplitudes, suggesting that an increased motoneuron excitability contributed to extra torques generation induced by the vibratory stimuli during electrically evoked contractions of the human calf muscles. The present study extends those previous results by showing that vibration alone may generate substantial levels of muscle force (around 20–25% of the maximal voluntary contraction (MVC)), with a centrally-mediated mechanism contributing to extra torque generation. Moreover, the soleus CMAP amplitudes are interpreted in the present paper as V/F-waves, as detailed above. Such responses may have also occurred in the previous study [51], even though at that time the authors interpreted them as F-waves exclusively.

It should be pointed out that the asymmetric behavior in force generation for a triangular-shaped muscle activation found in the present study is indeed a nonlinear phenomenon because the times involved in the increase and decrease in drive were very slow. This nonlinear behavior in force generation must certainly be taken into account by the central nervous system when planning and commanding specific muscle contractions and movements, probably by some previous learning of the specific behaviors shown by muscles.

The effect of vibration and exercise on human performance has been an issue of recent investigation [40,52-55]. However, the real effectiveness of vibration and the physiological mechanisms underlying the adaptive responses to vibration exercise remain controversial [56,57]. The results presented here suggest that vibratory stimuli, besides generating forces solely due to synaptic excitation (there is no direct stimulation of motor axons as may occur during electrically-evoked contractions), may trigger centrally-mediated excitatory mechanisms (e.g. plateau potentials). Thus, vibration-induced extra forces occurs in response to motoneuron recruitment presumably following the size principle, which is less fatiguing than forces generated by conventional patterns of electrical stimulation whose direct action on the motor axons either follows a random-order recruitment or a fast-fatigueable-first order of recruitment [58-60]. Therefore, vibratory stimulation may be suggested as a potential tool in a large diversity of rehabilitation protocols and exercise training programs. For example, vibration would be beneficial for therapeutic interventions designed to decrease muscle atrophy (in which the primary cause is the disuse-related loss of fatigue-resistant fibers [61]), or in rehabilitation protocols after spinal cord injury (in which paralyzed muscles often become more easily fatigued [62]).

## Conclusion

The results showed that vibratory stimuli (applied to the Achilles tendon) modulated in amplitude by triangular-shaped waves evoked increased forces during the decreasing phase of the vibration amplitude. A parallel increase in soleus V/F -wave amplitudes provided evidence that intrinsic neural mechanisms such as plateau potentials may play an important role in the extra torque generation. A similar mechanism (for example, induced by an excitatory descending drive rather than a vibratory stimulus) may assist in sustaining contractions during daily activities such as postural tasks or some types of voluntary movement. The vibration-induced increased motoneuron excitability (leading to increased torque generation) presumably activates spinal motoneurons following the size principle, which is a desirable feature for stimulation paradigms involved in rehabilitation programs and exercise training. The use of V-waves as indicators of the existence of spinal cord MNs that are discharging autonomously under the action of PICs is a methodological byproduct of the present research and may be a very useful tool for those studying these phenomena in humans.

### Endnote

^a^ In this paper, post-tetanic potentiation (PTP) refers to an increased synaptic transmission by the effects of a prior tetanus, causing PTP of the excitatory synaptic potential (caused by increased neurotransmitter release). In the literature, PTP is also used to describe the increase in force attributable to peripheral muscle properties, which will be named in this paper by their specific mechanisms such as myosin light chain phosphorylation.

## Competing interests

The authors declare that they have no competing interests.

## Authors’ contributions

FHM and AFK were equally involved in the conceptualization and design of the study. FHM and DRT recruited subjects, managed data collection, completed data analysis and drafted the manuscript. AFK supervised data collection, assisted with drafting and provided critical revision of the manuscript. All authors read and approved the final manuscript.

## References

[B1] Collins DF Burke D Gandevia SC Large involuntary forces consistent with plateau-like behavior of human motoneurons J Neurosci 2001 21 4059 4065 11356893 10.1523/JNEUROSCI.21-11-04059.2001 PMC6762712

[B2] Collins DF Burke D Gandevia SC Sustained contractions produced by plateau-like behaviour in human motoneurones J Physiol 2002 538 289 301 10.1113/jphysiol.2001.012825 11773336 PMC2290016

[B3] Bergquist AJ Clair JM Lagerquist O Mang CS Okuma Y Collins DF Neuromuscular electrical stimulation: implications of the electrically evoked sensory volley Eur J Appl Physiol 2011 111 2409 2426 10.1007/s00421-011-2087-9 21805156

[B4] Blouin JS Walsh LD Nickolls P Gandevia SC High-frequency submaximal stimulation over muscle evokes centrally generated forces in human upper limb skeletal muscles J Appl Physiol 2009 106 370 377 19008485 10.1152/japplphysiol.90939.2008

[B5] Nickolls P Collins DF Gorman RB Burke D Gandevia SC Forces consistent with plateau-like behaviour of spinal neurons evoked in patients with spinal cord injuries Brain 2004 127 660 670 14749290 10.1093/brain/awh073

[B6] Frigon A Thompson CK Johnson MD Manuel M Hornby TG Heckman CJ Extra forces evoked during electrical stimulation of the muscle or its nerve are generated and modulated by a length-dependent intrinsic property of muscle in humans and cats J Neurosci 2011 31 5579 5588 10.1523/JNEUROSCI.6641-10.2011 21490198 PMC4115248

[B7] Hyngstrom AS Johnson MD Miller JF Heckman CJ Intrinsic electrical properties of spinal motoneurons vary with joint angle Nat Neurosci 2007 10 363 369 10.1038/nn1852 17293858

[B8] Binder-Macleod S Kesar T Catchlike property of skeletal muscle: recent findings and clinical implications Muscle Nerve 2005 31 681 693 10.1002/mus.20290 15736271

[B9] Zhi G Ryder JW Huang J Ding P Chen Y Zhao Y Kamm KE Stull JT Myosin light chain kinase and myosin phosphorylation effect frequency-dependent potentiation of skeletal muscle contraction Proc Natl Acad Sci USA 2005 102 17519 17524 10.1073/pnas.0506846102 16299103 PMC1297671

[B10] Heckmann CJ Gorassini MA Bennett DJ Persistent inward currents in motoneuron dendrites: implications for motor output Muscle Nerve 2005 31 135 156 10.1002/mus.20261 15736297

[B11] Hultborn H Plateau potentials and their role in regulating motoneuronal firing Prog Brain Res 1999 123 39 48 10635702 10.1016/s0079-6123(08)62842-3

[B12] Kiehn O Eken T Functional role of plateau potentials in vertebrate motor neurons Curr Opin Neurobiol 1998 8 746 752 10.1016/S0959-4388(98)80117-7 9914232

[B13] Crone C Hultborn H Kiehn O Mazieres L Wigstrom H Maintained changes in motoneuronal excitability by short-lasting synaptic inputs in the decerebrate cat J Physiol 1988 405 321 343 3267152 10.1113/jphysiol.1988.sp017335 PMC1190978

[B14] Mendell LM Henneman E Terminals of single Ia fibers: location, density, and distribution within a pool of 300 homonymous motoneurons J Neurophysiol 1971 34 171 187 5540577 10.1152/jn.1971.34.1.171

[B15] Hounsgaard J Hultborn H Jespersen B Kiehn O Bistability of alpha-motoneurones in the decerebrate cat and in the acute spinal cat after intravenous 5-hydroxytryptophan J Physiol 1988 405 345 367 3267153 10.1113/jphysiol.1988.sp017336 PMC1190979

[B16] Bennett DJ Hultborn H Fedirchuk B Gorassini M Short-term plasticity in hindlimb motoneurons of decerebrate cats J Neurophysiol 1998 80 2038 2045 9772259 10.1152/jn.1998.80.4.2038

[B17] Bennett DJ Hultborn H Fedirchuk B Gorassini M Synaptic activation of plateaus in hindlimb motoneurons of decerebrate cats J Neurophysiol 1998 80 2023 2037 9772258 10.1152/jn.1998.80.4.2023

[B18] Fallon JB Macefield VG Vibration sensitivity of human muscle spindles and Golgi tendon organs Muscle Nerve 2007 36 21 29 10.1002/mus.20796 17471568

[B19] Roll JP Vedel JP Ribot E Alteration of proprioceptive messages induced by tendon vibration in man: a microneurographic study Exp Brain Res 1989 76 213 222 2753103 10.1007/BF00253639

[B20] Eklund G Hagbarth KE Normal variability of tonic vibration reflexes in man Exp Neurol 1966 16 80 92 10.1016/0014-4886(66)90088-4 5923486

[B21] Jankowska E McCrea D Mackel R Oligosynaptic excitation of motoneurones by impulses in group Ia muscle spindle afferents in the cat J Physiol 1981 316 411 425 6459446 10.1113/jphysiol.1981.sp013797 PMC1248803

[B22] Stuart GJ Rymer WZ Schotland JL Characteristics of reflex excitation in close synergist muscles evoked by muscle vibration Exp Brain Res 1986 65 127 134 3803498 10.1007/BF00243835

[B23] Gorassini MA Bennett DJ Yang JF Self-sustained firing of human motor units Neurosci Lett 1998 247 13 16 10.1016/S0304-3940(98)00277-8 9637398

[B24] Kamen G Sullivan R Rubinstein S Christie A Evidence of self-sustained motoneuron firing in young and older adults J Electromyogr Kinesiol 2006 16 25 31 10.1016/j.jelekin.2005.06.008 16099677

[B25] Kiehn O Eken T Prolonged firing in motor units: evidence of plateau potentials in human motoneurons? J Neurophysiol 1997 78 3061 3068 9405525 10.1152/jn.1997.78.6.3061

[B26] Birnbaum A Ashby P Postsynaptic potentials in individual soleus motoneurons in man produced by achilles tendon taps and electrical stimulation of tibial nerve Electroencephalogr Clin Neurophysiol 1982 54 469 471 10.1016/0013-4694(82)90211-5 6181970

[B27] Burke D Gandevia SC McKeon B The afferent volleys responsible for spinal proprioceptive reflexes in man J Physiol 1983 339 535 552 6887033 10.1113/jphysiol.1983.sp014732 PMC1199177

[B28] Burke D Gandevia SC McKeon B Monosynaptic and oligosynaptic contributions to human ankle jerk and H-reflex J Neurophysiol 1984 52 435 448 6090608 10.1152/jn.1984.52.3.435

[B29] Binder-Macleod SA Clamann HP Force output of cat motor units stimulated with trains of linearly varying frequency J Neurophysiol 1989 61 208 217 2918346 10.1152/jn.1989.61.1.208

[B30] Frigon A Carroll TJ Jones KE Zehr EP Collins DF Ankle position and voluntary contraction alter maximal M waves in soleus and tibialis anterior Muscle Nerve 2007 35 756 766 10.1002/mus.20747 17295303 PMC5005069

[B31] Zehr PE Considerations for use of the Hoffmann reflex in exercise studies Eur J Appl Physiol 2002 86 455 468 10.1007/s00421-002-0577-5 11944092

[B32] Sheskin DJ Handbook of Parametric and Nonparametric Statistical Procedures 2007 Boca Raton, Florida: Chapman & Hall/CRC Press

[B33] Cisi RR Kohn AF Simulation system of spinal cord motor nuclei and associated nerves and muscles, in a Web-based architecture J Comput Neurosci 2008 25 520 542 10.1007/s10827-008-0092-8 18506610

[B34] Elias LA Chaud VM Watanabe RN Kohn AF Application of a web-based simulator to a study of neuromuscular training in humans BMES 2011 2011 Hartford: BMES Society

[B35] Lin JZ Floeter MK Do F-wave measurements detect changes in motor neuron excitability? Muscle Nerve 2004 30 289 294 10.1002/mus.20110 15318339

[B36] Espiritu MG Lin CS Burke D Motoneuron excitability and the F wave Muscle Nerve 2003 27 720 727 10.1002/mus.10388 12766984

[B37] Salih F Steinheimer S Grosse P Excitability and recruitment patterns of spinal motoneurons in human sleep as assessed by F-wave recordings Exp Brain Res 2011 213 1 8 10.1007/s00221-011-2763-3 21717101

[B38] Giesebrecht S Martin PG Gandevia SC Taylor JL Altered corticospinal transmission to the hand after maximum voluntary efforts Muscle Nerve 2011 43 679 687 10.1002/mus.21938 21404298

[B39] Orhan EK Yayla V Cebeci Z Baslo MB Ovali T Oge AE Excitability changes at brainstem and cortical levels in blind subjects Clin Neurophysiol 2011 122 1827 1833 10.1016/j.clinph.2011.02.020 21454123

[B40] Christova M Rafolt D Mayr W Wilfling B Gallasch E Vibration stimulation during non-fatiguing tonic contraction induces outlasting neuroplastic effects J Electromyogr Kinesiol 2010 20 627 635 10.1016/j.jelekin.2010.03.001 20363152

[B41] Aagaard P Simonsen EB Andersen JL Magnusson P Dyhre-Poulsen P Neural adaptation to resistance training: changes in evoked V-wave and H-reflex responses J Appl Physiol 2002 92 2309 2318 12015341 10.1152/japplphysiol.01185.2001

[B42] Solstad GM Fimland MS Helgerud J Iversen VM Hoff J Test-retest reliability of v-wave responses in the soleus and gastrocnemius medialis J Clin Neurophysiol 2011 28 217 221 10.1097/WNP.0b013e31821215cf 21399516

[B43] Lee RH Heckman CJ Bistability in spinal motoneurons in vivo: systematic variations in rhythmic firing patterns J Neurophysiol 1998 80 572 582 9705451 10.1152/jn.1998.80.2.572

[B44] Heckman CJ Johnson M Mottram C Schuster J Persistent inward currents in spinal motoneurons and their influence on human motoneuron firing patterns Neuroscientist 2008 14 264 275 18381974 10.1177/1073858408314986 PMC3326417

[B45] Elias LA Chaud VM Kohn AF Models of passive and active dendrite motoneuron pools and their differences in muscle force control J Comput Neurosci 2012 33 515 531 10.1007/s10827-012-0398-4 22562305

[B46] Elias LA Kohn AF Individual and collective properties of computationally efficient motoneuron models of types S and F with active dendrites Neurocomputing 2013 99 521 533

[B47] Van Boxtel A Differential effects of low-frequency depression, vibration-induced inhibition, and posttetanic potentiation on H-reflexes and tendon jerks in the human soleus muscle J Neurophysiol 1986 55 551 568 3514814 10.1152/jn.1986.55.3.551

[B48] Fornari MC Kohn AF High frequency tendon reflexes in the human soleus muscle Neurosci Lett 2008 440 193 196 10.1016/j.neulet.2008.05.075 18555607

[B49] Knash ME Kido A Gorassini M Chan KM Stein RB Electrical stimulation of the human common peroneal nerve elicits lasting facilitation of cortical motor-evoked potentials Exp Brain Res 2003 153 366 377 10.1007/s00221-003-1628-9 14610631

[B50] Proske U Morgan DL Gregory JE Thixotropy in skeletal muscle and in muscle spindles: a review Prog Neurobiol 1993 41 705 721 10.1016/0301-0082(93)90032-N 8140258

[B51] Magalhaes FH Kohn AF Vibration-induced extra torque during electrically-evoked contractions of the human calf muscles J Neuroeng Rehabil 2010 7 26 10.1186/1743-0003-7-26 20537167 PMC2904788

[B52] Christova M Rafolt D Golaszewski S Gallasch E Outlasting corticomotor excitability changes induced by 25 Hz whole-hand mechanical stimulation Eur J Appl Physiol 2011 111 3051 3059 10.1007/s00421-011-1933-0 21455615

[B53] Fattorini L Ferraresi A Rodio A Azzena GB Filippi GM Motor performance changes induced by muscle vibration Eur J Appl Physiol 2006 98 79 87 10.1007/s00421-006-0250-5 16896736

[B54] Iodice P Bellomo RG Gialluca G Fano G Saggini R Acute and cumulative effects of focused high-frequency vibrations on the endocrine system and muscle strength Eur J Appl Physiol 2011 111 897 904 10.1007/s00421-010-1677-2 21063726

[B55] Barnes MJ Perry BG Mundel T 2011 Cochrane DJ: The effects of vibration therapy on muscle force loss following eccentrically induced muscle damage. Eur J Appl Physiol 10.1007/s00421-011-2064-3 21750975

[B56] Cardinale M Erskine JA Vibration training in elite sport: effective training solution or just another fad? Int J Sports Physiol Perform 2008 3 232 239 19208931 10.1123/ijspp.3.2.232

[B57] Rittweger J Vibration as an exercise modality: how it may work, and what its potential might be Eur J Appl Physiol 2010 108 877 904 10.1007/s00421-009-1303-3 20012646

[B58] Maffiuletti NA Minetto MA Farina D Bottinelli R Electrical stimulation for neuromuscular testing and training: state-of-the art and unresolved issues Eur J Appl Physiol 2011 111 2391 2397 10.1007/s00421-011-2133-7 21866361

[B59] Maffiuletti NA Physiological and methodological considerations for the use of neuromuscular electrical stimulation Eur J Appl Physiol 2010 110 223 234 10.1007/s00421-010-1502-y 20473619

[B60] Bickel CS Gregory CM Dean JC Motor unit recruitment during neuromuscular electrical stimulation: a critical appraisal Eur J Appl Physiol 2011 111 2399 2407 10.1007/s00421-011-2128-4 21870119

[B61] Gordon T Pattullo MC Plasticity of muscle fiber and motor unit types Exerc Sport Sci Rev 1993 21 331 362 8504847

[B62] Gerrits HL De Haan A Hopman MT van Der Woude LH Jones DA Sargeant AJ Contractile properties of the quadriceps muscle in individuals with spinal cord injury Muscle Nerve 1999 22 1249 1256 10.1002/(SICI)1097-4598(199909)22:9<1249::AID-MUS13>3.0.CO;2-N 10454722

